# Genetic Markers Are Associated with the Ruminal Microbiome and Metabolome in Grain and Sugar Challenged Dairy Heifers

**DOI:** 10.3389/fgene.2018.00062

**Published:** 2018-02-27

**Authors:** Helen M. Golder, Jennifer M. Thomson, Stuart E. Denman, Chris S. McSweeney, Ian J. Lean

**Affiliations:** ^1^Scibus, Camden, NSW, Australia; ^2^Dairy Science Group, School of Life and Environmental Sciences, Faculty of Science, The University of Sydney, Camden, NSW, Australia; ^3^Department of Animal and Range Sciences, Montana State University, Bozeman, MT, United States; ^4^Agriculture and Food (CSIRO), St. Lucia, QLD, Australia

**Keywords:** fructose, genome-wide association, lactic acid, matrix extracellular phosphoglycoprotein, pleiotropy, ruminal microbiome, ruminal acidosis

## Abstract

Dairy heifers were subjected to a non-life-threatening challenge designed to induce ruminal acidosis by feeding grain and sugar. Large among animal variation in clinical signs of acidosis, rumen metabolite concentrations, and the rumen microbiome occurred. This exploratory study investigates sources of the variation by examining associations between the genome, metabolome, and microbiome, albeit with a limited population. The broader objective is to provide a rationale for a larger field study to identify markers for susceptibility to ruminal acidosis. Initially, heifers (*n* = 40) allocated to five feed additive groups were fed 20-days pre-challenge with a total mixed ration and additives. Fructose (0.1% of bodyweight/day) was added for the last 10 days pre-challenge. On day 21 heifers were challenged with 1.0% of bodyweight dry matter wheat + 0.2% of bodyweight fructose + additives. Rumen samples were collected via stomach tube weekly (day 0, 7, and 14) and at five times over 3.6 h after challenge and analyzed for pH and volatile fatty acid, ammonia, D-, and L-lactate concentrations. Relative abundance of bacteria and archaea were determined using Illumina MiSeq. Genotyping was undertaken using a 150K Illumina SNPchip. Genome-wide association was performed for metabolite and microbiome measures (*n* = 33). Few genome associations occurred with rumen pH, concentration of acetate, propionate, total volatile fatty acids, or ammonia, or the relative abundance of the Firmicutes, Bacteroidetes, and Spirochaetes phyla. Metabolites and microbial phyla that had markers associated and quantitative trait loci (QTL) were: acetate to propionate ratio (A:P), D-, L-, and total lactate, butyrate, acidosis eigenvalue, Actinobacteria, Chloroflexi, Euryarchaeota, Fibrobacteres, Planctomycetes, Proteobacteria, and Tenericutes. A putative genomic region overlapped for Actinobacteria, Euryarchaeota, and Fibrobacteres and covered the region that codes for matrix extracellular phosphoglycoprotein (MEPE). Other overlapping regions were: (1) Chloroflexi, Tenericutes, and A:P, (2) L- and total lactate and Actinobacteria, and (3) Actinobacteria, Euryarchaeota, Fibrobacteres, and A:P. Genome-wide associations with the metabolome and microbiome occurred despite the small population, suggesting that markers for ruminal acidosis susceptibility exist. The findings may explain some of the variation in metabolomic and microbial data and provide a rationale for a larger study with a population that has variation in acidosis.

## Introduction

There is interest in understanding the links between the mammalian genotype and the closely associated enteric biome. Along with the influence of these associations on disease. Ruminal acidosis is a complex nutritional disorder of worldwide concern. Estimates of the prevalence in dairy cattle are between 10 and 26.7% (Bramley et al., 2008; Tajik et al., 2009) and feedlot cattle and sheep are also at high risk of ruminal acidosis. The condition is associated with an accumulation of organic acids within the rumen initiated by the combination of consumption of large amounts of readily fermentable carbohydrates and insufficient intake of physically effective fiber (Nagaraja and Titgemeyer, 2007; Bramley et al., 2008). It can be difficult to achieve a balance between feeding readily fermentable carbohydrates to meet the high energy demands required for productivity and to minimize adverse health risks that result from feeding starches and sugars. Several studies have noted considerable variation in responses among cattle fed a common diet designed to induce ruminal acidosis (Brown et al., 2000; Bevans et al., 2005; Penner et al., 2009; Golder et al., 2014a). Current control strategies are unlikely to manage ruminal acidosis in the entire herd due to this considerable variation (Golder et al., 2014a).

The review by Firkins and Yu (2015) notes that differences in the microbiome structure among animals within the same diet group often exceeds those among different diet groups. These differences have been explained by different host genetics and interactions with the rumen microbiome (Weimer et al., 2010; King et al., 2011; Hernandez-Sanabria et al., 2013). As suggested in humans and mice (Benson et al., 2010), bovines appear to have a core microbiome that differs among individuals (Li et al., 2009; Welkie et al., 2010; Jami and Mizrahi, 2012; Henderson et al., 2015). It has not been established if this core microbiome is the result of genetics, environment, or their interaction, or how this may influence susceptibility to ruminal acidosis or other disorders. A mechanistic understanding of gut function and gut microbiota is relevant to our understanding of acidosis in ruminants as well as other disease conditions and production outcomes. Currently, no data are available in the Animal quantitative trait loci (QTL) database for rumen parameters or microbial composition. Genetic selection for desirable traits has improved production and welfare in several animal industries including cattle; therefore, identification of markers linked to favorable rumen conditions could lead to genetic selection toward more acidosis resistant and productive cattle.

The aim of this work was to examine associations between the bovine genome, metabolome, and microbiome, hence to investigate the large among animal variation observed in cattle fed feed additive interventions and subjected to a non-life-threatening, but substantial, starch and fructose challenge (Golder et al., 2014a). The objective is to use this exploratory work to provide a rationale for a larger field study where markers for the susceptibility of ruminal acidosis may be identified for possible use in breeding decisions. We hypothesized that associations would occur between the bovine genome, metabolome, and microbiome and QTL would be identified.

## Materials and methods

This study was carried out in accordance with the recommendations of The Australian Code for Care and Use of Animals for Scientific Purposes, S*cibus* Animal Ethics Committee. The protocol was approved by the S*cibus* Animal Ethics Committee (S*cibus* 0512-0513 and 0414-0714).

**Figure 1:**
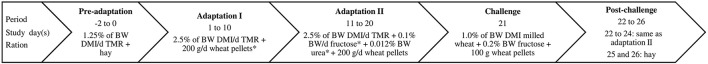
Experimental periods and their corresponding study days and rations offered during the study. The rations were offered in equal proportions twice daily, with the exception of the challenge period. Rumen samples were collected on day 0, 7, 14, and 21 during their respective experimental periods. Wheat pellets contained respective feed additives for their groups as indicated in Table 1. Heifers in the monensin + live yeast group received yeast and those in the sodium bicarbonate + magnesium oxide group received sodium bicarbonate and magnesium oxide in addition to wheat pellets. ^*^Introductory doses were offered for the initial days before the full rate was offered. BW, bodyweight; DMI, dry matter intake; TMR, total mixed ration; hd, head; d, day (62:38 forage:concentrate, consisting of 31.5% wheaten hay, 30.5% alfalfa hay, and 38% milled wheat. Reprinted from Golder et al. (2014a) with permission from Elsevier.

Phenotypic and genotypic data were obtained from 36 pregnant and 4 non-pregnant Holstein heifers. These heifers were sourced from a commercial Holstein herd that primarily used an artificial insemination breeding program. These heifers were enrolled in a study to investigate the efficacy of feed additives during a starch and fructose challenge. The experimental design and diets are described in detail by Golder et al. (2014a). Briefly, all heifers were between 15 and 21 months of age and a mean bodyweight (BW) of 383 ± 49 kg on arrival at the study site located at Cobbitty, New South Wales (NSW), Australia. Each heifer was enrolled in the study for a period of 29-days consisting of 5 experimental periods: (1) preadaptation (days −2 to 0), (2) adaptation I (days 1–10), (3) adaptation II (days 11–20), (4) challenge (day 21), and (5) post-challenge (days 22–26; Figure 1). The preadaptation, adaptation I, and adaptation II periods are often referred to collectively as the pre-challenge periods. The preadaptation period was designed to allow collection of baseline samples and acclimatize the heifers to the study pens and a total mixed ration. The adaptation I period was designed to adapt the heifers' rumen microbiota to the feed additives, while the adaptation II period was designed to examine the influence of fructose on the microbiota and the efficacy of the feed additives. For the duration of the study all heifers, when not being fed or sampled, were kept as one herd in a paddock with little or no available feed and with *ad libitum* water access. Each heifer was allocated to one of five feed additive groups (*n* = 8 heifers/feed additive group) that are detailed in Table 1. The efficacy of feed additives to reduce acidosis risk during a non-life-threatening, but substantial, starch and fructose challenge was assessed to allow producers, nutritionists, and veterinarians to make informed management decisions when considering use of feed additives and assist in the development of the most prudent use strategies for antimicrobial and other agents that modify rumen function (Golder et al., 2014a). Heifers were fed twice daily at approximately 07:00 and 14:00 h a 62% forage:38% concentrate total mixed ration at 1.25% of BW dry matter/day for a 20-day adaptation period with their additive(s) in individual concrete floor feeding pens. Pens were cleaned between heifers and feeding sessions to avoid cross-contamination of feed additives. Fructose (0.1% of BW/day) was added to the ration for the last 10 days of pre-challenge (adaptation II). On day 21 heifers were challenged with a ration consisting of 1.0% of BW dry matter milled wheat and 0.2% of BW fructose plus their additive(s). The challenge ration had an estimated non-fiber carbohydrate content of 76.3% of dry matter (CPM Dairy ration Analyzer, Cornell University, Ithaca, NY, USA). Hence, was designed to provide a substantial but non-life-threatening acidosis challenge to test the efficacy of the feed additives.

**Table 1:** Feed additives administered to each feed additive group. Reprinted from Golder et al. (2014a) with permission from Elsevier.

**Group**	**Feed additives**					
	**Active constituent**	**Commercial name**	**Manufacturer**	**Delivery form**	**Active (mg)**	**Dose rate (g/head/day)**
Control^a^	–	–	–	–	–	–
Virginiamycin	Virginiamycin	Eskalin	Phibro Animal Health, Girraween, New South Wales, Australia	Pellet^b^	200	10
Monensin + tylosin	Sodium monensin Tylosin	Rumensin 100 Tylan	Elanco Animal Health, West Ryde, Australia Elanco Animal Health	Pellet containing both additives^b^	200 110	2.2 0.44
Monensin + live yeast	Sodium monensin *Saccharomyces cerevisiae* CNCM I-1077	Rumensin 100 Levucell SC Direct	Elanco Animal Health Lallemand Animal Nutrition, Maroochydore, Queensland, Australia	Pellet^b^ Dry active yeast	220 500^c^	2.5 25
Sodium bicarbonate + magnesium oxide^a^	Sodium bicarbonate Magnesium oxide	Sodium bicarbonate Causmag	Penice Soda Products Pty Ltd, Osborne, South Australia, Australia Causmag International, Young, New South Wales, Australia	Powder Fine granules (mean particle size 0.85 mm)	- -	200 30

a *Wheat pellets were given containing no feed additives*.

b *Pellets comprised respective feed additives, disc milled wheat and 2.5 g/head/day of mineral premix (Cows R Us Base, DSM Nutritional Products, Wagga Wagga, Australia) and were pelleted using a cold pellet press*.

c *10 billion coliform forming units (CFU)/head/day*.

### Phenotypic data

Phenotypic data included both metabolomic and microbial data. The metabolomic measures included: total VFA, acetate, butyrate, propionate, valerate, ammonia, total lactate, D-lactate, and L-lactate concentrations, ratio of acetate to propionate (A:P), pH, and acidosis eigenvalues. The predicted means (±SEM) for these measures at each period of the study for each feed additive group are provided in Golder et al. (2014a). The microbial data included the relative abundance of the bacterial phyla: Actinobacteria, Bacteroidetes, Chloroflexi, Fibrobacteres, Firmicutes, Planctomycetes, Proteobacteria, Spirochaetes, and Tenericutes and the archaeal phylum, Euryarchaeota. The raw means (±*SD*) for these data for each feed additive group are shown in Table 2 for rumen fluid collected 215-min after consumption of the challenge ration.

**Table 2:** Group raw means (±*SD*) for the relative abundance of ruminal bacterial and archaeal phyla from rumen fluid collected 215 min after challenge ration consumption.

**Bacterial phyla (relative abundance)**	**Feed additive group**				
	**Control (no feed additives)**	**Virginiamycin**	**Monensin+tylosin**	**Monensin+live yeast**	**Buffers**
Actinobacteria	0.50 ± 0.57	0.39 ± 0.38	1.16 ± 1.04	0.85 ± 0.88	1.15 ± 1.88
Bacteroidetes	50.85 ± 22.08	64.44 ± 20.39	60.81 ± 11.65	67.30 ± 12.00	46.66 ± 20.78
Chloroflexi	0.96 ± 0.91	0.38 ± 0.42	0.74 ± 1.53	0.16 ± 0.38	1.16 ± 1.36
Fibrobacteres	0.71 ± 0.51	0.91 ± 0.87	0.26 ± 0.52	0.38 ± 0.48	0.56 ± 0.57
Firmicutes	40.50 ± 23.92	28.14 ± 15.57	32.06 ± 10.49	27.65 ± 11.13	41.20 ± 18.11
Planctomycetes	0.33 ± 0.30	0.23 ± 0.24	0.34 ± 0.47	0.06 ± 0.18	0.34 ± 0.34
Proteobacteria	0.51 ± 0.43	0.20 ± 0.19	1.09 ± 0.93	0.94 ± 1.15	2.78 ± 7.16
Spirochaetes	3.06 ± 3.05	3.74 ± 3.53	1.87 ± 1.63	1.25 ± 1.25	3.23 ± 3.03
Tenericutes	1.95 ± 1.12	1.00 ± 0.75	1.19 ± 1.00	1.03 ± 0.52	1.84 ± 1.93
**Archaeal phylum (relative abundance)**					
Euryarchaeota	0.41 ± 0.41	0.15 ± 0.24	0.24 ± 0.36	0.20 ± 0.23	0.86 ± 1.44

### Metabolomic data

Rumen samples were collected weekly (days 0, 7, and 14) and at 5-time points over 3.6 h after consumption of the challenge ration (day 21) providing a total of 8 values per phenotypic measure (One from the preadaptation, adaptation I, and adaptation II periods and 5, 65, 115, 165, and 215 min after the consumption of the challenge diet on challenge day; Figure 1). Rumen fluid was collected using a custom-designed stomach tube and scored for saliva contamination using the 3-point system described by Bramley et al. (2008). The rumen fermentation measures were analyzed according to methods described by Golder et al. (2014a). The acidosis eigenvalues are designed to provide an indication of the degree of ruminal disturbance or ruminal acidosis as a value between zero and one (zero being a healthy rumen and one being acidotic). The acidosis eigenvalues were originally derived from the work of Bramley et al. (2008) and are based on a discriminant analysis of standardized VFA, pH, ammonia, and lactate analyses. Effectively, they describe how closely the rumen sample analysis from a heifer from the current study matches the centroid of the acidotic cattle from Bramley et al. (2008). The model generated by Bramley et al. (2008) did not contain cattle with high lactic acid concentrations; therefore, acidosis eigenvalues are not validly generated for populations with large amounts of lactate present in rumen samples. As a result, acidosis eigenvalues were only calculated for the pre-challenge periods.

### Microbial data

The rumen fluid used for microbial analysis was collected at the same time as that collected for analysis of rumen fermentation measures by Golder et al. (2014a) but was not sieved after collection. These unprocessed samples that contained both liquid and solid phases were immediately aliquoted into 1.5 mL collection tubes and placed in liquid nitrogen prior to storage at −80°C until microbial processing.

#### DNA extraction

Ruminal fluid samples (*n* = 320) were thawed at room temperature and a 1 mL aliquot was centrifuged at 15,000 × *g* for 5-min and the supernatant discarded. The pellet was resuspended by vigorous vortexing and disturbance with a pipette tip in 1 mL of RBB+C lysis buffer and 200 mg of 0.1 mm silica-zirconium beads (Daintree Scientific, St Helens, TAS, Australia) was added. The mixture was homogenized in a Tissuelyser II (Qiagen GmbH, Hilden, North Rhine-Westphalia, Germany) at 30 cycles/s for 2-min, rotating 180° at 1 min, heated at 70°C for 15 min, and spun at 15,000 × *g* for 5-min. Supernatant was removed and 200 μL of AL buffer (QIAmp DNA mini kit cat no. 51306; Qiagen GmbH) was added. Mixture was incubated at 56^o^C for 10-min before addition of 200 μL of 100% ethanol and vortexing for 15 s. Mixture was transferred to a QIAmp mini spin column (QIAmp DNA mini kit) and centrifuged at 16,000 × *g* for 1-min. Filtrate was discarded and 500 μL of AW1 buffer (QIAmp DNA mini kit) was added to the column that was placed in a new 2 mL collection tube and centrifuged at 16,000 × *g* for 1-min. This step was repeated twice with AW2 buffer (QIAmp DNA mini kit). The column was dried by centrifugation at 16,000 × *g* for 1-min before addition of 100 μL of AE buffer (QIAmp DNA mini kit) and incubation of room temperature for 2-min. DNA was eluted by centrifugation at 16,000 × *g* for 1-min into a 1.5 mL collection tube. DNA was quantified using a Nanodrop spectrophotometer ND-1000 (Thermo Scientific, Waltham, MA, USA).

#### PCR amplification of 16S ribosomal DNA gene sequences

The QIAmp DNA mini kit cat no. 51306 (Qiagen GmbH, Hilden, North Rhine-Westphalia, Germany) was used for DNA extraction. The 16S rRNA gene spanning the V4 region was PCR amplified from genomic DNA from each sample using Platinum taq polymerase (Life Technologies Australia Pty. Ltd., Mulgrave, VIC, Australia) as follows: 1 cycle at 94°C for 1-min; followed by 25 cycles of 94°C for 5-s, 55°C for 30-s, 68°C for 45-s, with a final extension of 68°C for 7-min. Primers were those described by Kozich et al. (2013). In addition, a unique 8 base pair barcode was included in the reverse primer of each amplicon, so that DNA sequence reads can be assigned accurately to each originating sample. The PCR products were visualized on agarose gels and equal amounts of PCR product were pooled and gel extracted (Qiaex II gel extraction kit cat no. 20021, Qiagen GmbH). A total of ~10 ng/μL of pooled amplicon was sent to Macrogen (Seoul, South Korea) for sequencing using the Illumina® MiSeq platform according to manufacturer's instructions.

#### Sequence analyses of gene amplicons

Sequence data was analyzed using the Quantitative Insights Into Microbial Ecology software package (QIIME; Caporaso et al., 2010). Sequences were assigned to their originating sample based on the attached barcode and filtered based on quality and length parameters. Error correction was performed using Acacia (Bragg et al., 2012). Clustering of sequences to an operational taxonomic unit at a 0.97 distance threshold performed using uclust (Edgar, 2010). Chimeric sequences were identified using chimera slayer (Haas et al., 2011) and removed. Taxonomic identification was based on similarity to the Greengenes Database (http://greengenes.lbl.gov) using RDP classifier software (Wang et al., 2007). Only bacterial phyla with mean relative abundances of >0.3% in at least one of the five feed additive treatment groups at any of the study periods were included in the phenotypic data.

### Genotypic data

Blood was collected by tail venipuncture in blood tubes containing no anticoagulant from 27 of the cattle 2 years after the initial experiment in which the rumen fluid was collected. Whole blood was frozen at −20°C until analysis. For 13 of the cattle that no-longer remained in the herd serum samples stored at −20°C that had been processed from blood collected by tail venipuncture during the initial experiment were used. There was either not enough sample or the extracted DNA was of too low a quality from six of the heifers.

#### DNA extraction

Bovine DNA from frozen whole blood samples was extracted using Maxwell 16 LEV Blood DNA Kit Cat no. AS1290 (Promega, Madison, WI, USA) and Maxwell 16 Semi Automated Nucleic Acid Extractor (Promega) according to manufacturer recommended procedures and 30 μL of DNA elutant was frozen at −20°C until further analysis.

Bovine DNA from frozen serum samples were also extracted using Maxwell 16 LEV Blood DNA Kit Cat no. AS1290 and Maxwell 16 Semi Automated Nucleic Acid Extractor (Promega). Instead of blood preparation the following modification was run. Approximately 5 mL of vortexed, room temperature serum was poured through a 5 μm filter to capture intact cells and cellular debris. The filter was then washed twice with 150 μL of sterile nuclease free water. The material washed from the filter and suspended in water was then added to 1.5 mL micro-centrifuge tubes and 30 μL of proteinase K and 300 μL of lysis buffer was added. Tubes were then sealed and placed in a 56°C heating block for 20-min. After incubation, Maxwell 16 LEV blood cartridges (Promega), were prepared and blood lysate was transferred to well number one of each cartridge. The elution tubes were placed in the LEV rack with each cartridge and 30 μL of elution buffer was placed in each elution tube. The rack containing cartridges and elution tubes was run in a Maxwell 16 Semi Automated Nucleic Acid Extractor (Promega). Once the run was complete, elution tubes containing DNA were frozen at −20°C until further analysis.

Initial DNA quality and quantity was determined by reading the absorbance at 260 and 280 nm frequencies. Samples yielding a ratio between 1.65 and 1.95 were considered to have sufficient purity for genotyping and samples that yielded at least a DNA concentration of 10 ng/μL. Blood samples averaged 30 ng/μL and serum samples averaged 12 ng/μL.

When necessary, additional purification was carried out utilizing 1 mL of neutralized phenol/chloroform/iso-amyl alcohol (25:24:1). Samples were then mixed vigorously and allowed to sit for 1 h. They were then spun at 10,000 *g* in a microfuge for 5-min. The upper phase was transferred to a new microfuge tube and then pipetted several times to break up the gelatinous mass. Ethanol (1 mL of 100%) was added and inverted until DNA precipitate formed. The sample was then spun in a microfuge again for 5-min and supernatant discarded. The tube was then subjected to a quick spin and the last drop of ethanol removed with a 25 μL capillary tube. The samples were air dried at room temperature overnight. Tris-EDTA buffer (100 μL) was added and samples were incubated at 65°C for 15-min to re-suspend DNA.

#### Genotyping

All DNA samples of sufficient quality and quantity were shipped on ice to Neogen (Lincoln, NE, USA) for analysis using the Geneseek Genomic Profiler Bovine 150K Illumina SNPchip. Chips were prepared and run according to manufacturer guidelines.

The single-nucleotide polymorphism (SNP) genotypes by animal identification, SNP mapping file containing chromosome and position for each SNP, and phenotype by animal identification files were imported into Golden Helix software (Bozeman, MT, USA).

#### Data quality control

Single-nucleotide polymorphisms were dropped from the analysis if call rate was <0.9 and minor allele frequency was <0.01. Hardy-Weinberg Equilibrium was calculated and SNPs were dropped if Fisher's exact test for Hardy-Weinberg Equilibrium *P*-value was < 0.0001. After quality control filtering 125,829 SNPs were included in analysis.

Linkage disequilibrium pruning was performed with a window size of 100 and a window increment of 5. The linkage disequilibrium *r*^2^ threshold was 0.5 and composite haplotype method for the Linkage disequilibrium computation method. This inactivated 29,843 markers and left 95,986 in the analysis. Principal component analysis was used to account for population stratification as sire and dam identifications were not known.

### Statistical analysis

#### Genome-wide association analysis

Genome-wide association testing was completed on data from individual phenotypes collected at the 8 sampling time points during the study (One from the preadaptation, adaptation I, and adaptation II periods and 5, 65, 115, 165, and 215 min after the consumption of the challenge diet on challenge day). If > eight significant markers were identified from any of these time points, all significant markers were combined from the eight phenotype tests for QTL analysis.

Genome-wide association testing was completed using an additive model (dd) -> (Dd) -> (DD) and linear regression where the response *y* was fit to every genetic predictor variable or encoded genotype. The response was represented with the formula *y* = *b*_1_*x* + *b*_0_ + ∈, where the model was represented by the expression *b*_1_*x* + *b*_0_ and the error term, ε, expressing the difference, or residual, between the model of the response and the response itself (BuŽková, 2013). This model was chosen for its simplicity and ease of understanding as this was designed as an exploratory experiment and in an attempt to avoid adding bias to the results. A Bonferroni adjustment was applied, this adjustment multiplies each individual *P*-value by the number of genotype predictor values being tested. This value seeks to estimate and control for the probability that the association test would have obtained the same value by chance and is a conservative method for accounting for false discovery rate (Sham and Purcell, 2014). Population stratification was corrected for using Principal component analysis using the method of the program EIGENSTRAT as described by Price et al. (2006). This methodology was applied for this exploratory analysis as little to no information was available on animal genetic variance related to the phenotypic characteristics studied.

Positional candidate genes were identified in genomic regions with multiple significant associations or around markers that were significantly associated to a phenotype over multiple measurement periods. A window of 100,000 base pairs was used in the case of a single associated marker and where clusters of markers were identified the positional candidate genes were identified within a window of three times the span of the cluster. Associations were conducted utilizing individual animal phenotypes rather than grouping the phenotypes by feed additive group to avoid bias in genetic associations.

#### Spearman rank correlations

Spearman rank correlations were performed using GenStat (14th edition; VSN International Ltd., Hemel Hempstead, UK) between rumen metabolite measures and the relative abundance of rumen microbial taxa that had associated regions. Correlations were only performed for the sampling time points where these associations occurred.

## Results

Bovine DNA was successfully extracted from 25 whole blood samples and 9 serum samples. A heifer from the monensin + tylosin group who had a successful DNA extraction from whole blood was removed as she consumed only 12% of her challenge ration, leaving a total of 33 heifers in the analysis. Thirty one of these 33 heifers were pregnant.

### Host-metabolome interactions

In general, the association analysis found a greater number of significant associations at the pre-challenge samplings. Table 3 shows that few markers were significantly associated with total rumen VFA concentration or with the concentrations of specific VFA. There were 0, 32, 1, 39, and 30 markers associated with the A:P at the five samplings over a 3.6-h period on challenge day. A greater number of associations were found with total lactate (88, 97, and 8) during the pre-challenge period and a similar pattern was observed with the concentrations of D-lactate and L-lactate. There was little relationship between host genotype and rumen pH. The acidosis eigenvalue had 20, 5, and 18 significantly associated markers (Adjusted *P*-value < 0.05) during the pre-challenge period.

**Table 3:** Number of significant markers (Adjusted *P*-value < 0.05) for rumen and bacterial and archaeal phyla phenotypes from rumen fluid collected at 7-day intervals, Pre, preadaptation (day 0); AI, adaptation I (day 7); AII, adaptation II (day 14); and challenge day (day 21).

**Phenotype**	**Pre-challenge**			**Challenge**				
	**Pre**	**AI**	**AII**	**5**	**65**	**115**	**165**	**215**
**Rumen measures**								
Total VFA (m*M*)	0	0	1	1	1	0	0	0
Acetate (m*M*)	0	0	2	1	1	0	0	0
Propionate (m*M*)	1	0	0	0	0	0	0	0
Acetate:Propionate	0	0	1	0	32	1	39	30
Butyrate (m*M*)	0	10	0	0	2	3	2	1
Valerate (m*M*)	0	0	10	0	0	28	2	0
Ammonia (m*M*)	0	0	1^*^	0	0	0	0	0
Total lactate (m*M*)	88	97	8	0	0	1	0	0
D-lactate (m*M*)	90	10	24	0	2	0	1	1
L-lactate (m*M*)	81	102	4	13	0	0	0	0
pH	1^*^	0	2	0	2	0	2	0
Acidosis eigenvalue^a^	20	5	18	–	–	–	–	–
**Bacterial phyla (relative abundance)**								
Actinobacteria	NA	129	34	3	93	3	19	2
Bacteroidetes	1	1^*^	1	0	0	1	1	1^*^
Chloroflexi	1	27	3	2	5	10	3	62
Fibrobacteres	0	21	22	1	67	6	0	0
Firmicutes	1	1	1	0	0	0	1	0
Planctomycetes	9	0	2	6	0	1	0	1
Proteobacteria	0	14	36	30	2	51	42	35
Spirochaetes	0	3	0	0	1	5	1	3
Tenericutes	0	19	0	2	1^*^	2^*^	7	29
**Archaeal phylum (relative abundance)**								
Euryarchaeota	7	6	0	0	16	27	96	46

*On the challenge day, rumen fluid samples were collected 5, 65,115, 165, and 215 min after challenge ration consumption*.

* *Tendency only, adjusted P-value between 0.05 and 0.1*.

a *Derived from discriminant analysis of standardized values for rumen acetate, propionate, butyrate, valerate, iso-butyrate, iso-valerate, ammonia, pH, and D-lactate (Bramley et al., 2008)*.

Putative QTL were identified for several metabolite phenotypes (Supplementary Table 1). Five putative QTL were identified for the A:P on chromosome 1, 3, 5, 6, and 8. Eight putative QTL regions were identified for total lactate concentrations on chromosomes 1, 4, 6, 11, 22, and 24. Three putative QTL regions were identified for D-lactate concentrations on chromosomes 2, 8, and 26 and six putative QTL were identified for L-lactate concentrations on chromosomes 1, 4, 8, 17, and 24. One QTL region was identified for butyrate concentration on chromosome 16. One QTL was identified for the acidosis eigenvalue on chromosome 19. A QTL region on chromosome 1, 4, and 24 was similar for total lactate and L-lactate. No regions were similar for total lactate and D-lactate.

### Host-microbiome interactions

The association analysis could not be conducted for Actinobacteria during the preadaptation time point because no Actinobacteria were detected at this sampling. Few markers were significantly associated with the relative abundance of Bacteroidetes, Firmicutes, or Spirochaetes at either the pre-challenge or challenge measurements as summarized in Table 3. A number of associations were observed at more than one-time point for the relative abundance of Chloroflexi, Fibrobacteres, Firmicutes, Planctomycetes, Proteobacteria, Tenericutes, and Euryarchaeota (Table 3). The largest number of associations occurred for the relative abundance of Actinobacteria at adaptation I, followed by the relative abundance of Euryarchaeota at the 165-min sampling point.

Putative QTL were identified for several microbial phyla (Supplementary Table 2). Ten putative QTL regions were identified for Actinobacteria with significant markers found over multiple measurements. These include regions on chromosomes 1, 2, 3, 4, 6, 8, 14, 16, 22, and 24. Two putative QTL regions were identified for Chloroflexi on chromosomes 1 and 3. One QTL region was identified for Fibrobacteres on chromosome 6. One putative QTL region was identified for Planctomycetes on chromosome 15. Twenty-one putative QTL regions or markers were identified for Proteobacteria on chromosomes 1, 3, 4, 5, 6, 7, 8, 10, 12, 17, 18, 25, 26, 27, and 29. Two putative QTL regions were identified for Tenericutes on chromosome 1 and 15. Six putative QTL regions were identified for Euryarchaeota on chromosomes 6, 10, 13, and 20. One region on chromosome 1 over-lapped for Chloroflexi and Tenericutes. One region on chromosome 6 overlapped between Actinobacteria, Euryarchaeota, and Fibrobacteres and encompassed the region that codes for *MEPE*, matrix extracellular phosphoglycoprotein (MEPE).

Additionally, three regions demonstrated pleiotropy with overlap between the region shared by Chloroflexi and Tenericutes with the region associated with rumen A:P on chromosome 1. A positive Spearman rank correlation occurred between the abundance of Chloroflexi and Tenericutes at the overlapping sampling point and had a *t*-value of 0.050; while, *t*-values for the positive correlations between Tenericutes and A:P and Chloroflexi and A:P were >0.05. Another region that was associated with both microbial phyla and rumen metabolites is on chromosome 4 and overlapped for total lactate and L-lactate as well as Actinobacteria. Spearman rank correlations from the overlapping time point indicated that L- and total lactate concentrations were highly positively correlated with a *t*-value of < 0.001, but positive correlations between L-lactate and Actinobacteria and total lactate and Actinobacteria were not significant. Similarly, the region of chromosome 6 associated with Actinobacteria, Euryarchaeota, and Fibrobacteres overlaps a region significantly associated with the rumen A:P. These regions were not identified from a consistent time point.

## Discussion

Associations between the bovine genome, metabolome, and microbiome and identification of QTL, as hypothesized, can be related to physiological challenge and suggest that some of the variation in responses in microbial data (Table 2) and metabolomic data that were observed previously (Golder et al., 2014a) may be attributed to the host genome. The study design used in the initial animal experiment (Golder et al., 2014a) from which the data for this current work is derived was not initially intended for use to explore genomic relationships; hence, the study population in the current work is not ideal for genomic analysis. Typically, a larger population size, evaluated at the same physiological status, and fed an identical diet would be used. However, with the limited data currently available in this field and the conservative nature of the statistical approach used, this work provides indications for future directions. These findings provide a strong rationale for a subsequent study with a larger number of animals.

Benson et al. (2010) used a similar approach to our study utilizing a mouse model, in which, they examined factors that influence microbial composition in an intercross line (*n* = 645). They determined that family/litter (10%) and cohort (26%) had significant impacts on gut microbiota composition which left host genetics to play a major role in explaining the remaining variation. Benson et al. (2010) used a set of 530 markers, while the present study utilized a final filtered set of approximately 95,000 markers, but in a smaller number of animals (33). As in the present study, Benson et al. (2010) reported that microbial phyla behaved like polygenic traits with multiple QTL reported for each phylum and additional QTL identified at the level of family and genus. As this current work is only exploratory, family and genera were not investigated but are intended to be explored in a larger field study. Similar to the current study, Benson et al. (2010) also observed pleiotropy with several QTL that appeared to be relevant on multiple taxa. The relationship among microbial phyla, host genome, and metabolite, specifically for the region on chromosome 4 that had overlapping putative QTL for concentration of total lactate and L-lactate and for relative abundance of Actinobacteria, which have been previously associated with subacute ruminal acidosis (Mao et al., 2013), provides support for the approach taken in this study. Further, the identification of interesting positional candidate genes such as MEPE in regions associated with the relative abundance of Actinobacteria, Euryarchaeota, and Fibrobacteres may provide novel directions for future research.

Benson et al. (2010) demonstrated in murine models, that the gut microbiota can now be viewed as an environmental factor that itself is controlled in part by host genetic factors and potentially by interactions between host and microbial genomes. This view implies that genetic predisposition to complex diseases may be manifested in part by a predisposition to aberrant patterns of microbial colonization, which in turn contribute to disease processes (Benson et al., 2010).

We recognize that a larger study with different diets and expression of acidosis will provide more insights to the associations studied. Factors that may have influenced our responses include; the exposure of cattle to feed additives; a limited opportunity for the microbiomes to adapt to the three ration changes; inherent variability among individuals in the time that the microbiome adapts to diets; and pregnancy status and stage of pregnancy. The differences in number of associations identified for each of the phenotypes between the pre-challenge and challenge periods highlights the importance of exploring genomic relationships at each of the 8-time points and suggests an influence of diet. Interpretations of these differences are limited by the low population size, but it is important to note that the associations and QTL identified for D-, L-, and total lactate concentration occurred pre-challenge, coinciding with lower among animal variation; whereas, associations for A:P and valerate concentration occurred from samples collected on the Challenge day.

Our findings that suggest responses to rumen disturbances could be host specific, as indicated by the associations between the host genome, metabolome, and microbiome support those of Weimer et al. (2010). These authors showed the ability of the rumen to revert to pre-exchange VFA concentration and rumen pH and nearly return to pre-exchange bacterial community composition within 24-h of a 95% exchange of ruminal content with a cow on a similar diet. A second cow took a longer period to revert indicating the potential for variability in this response (Weimer et al., 2010). While Chen et al. (2012) discussed possible links between acidosis and immune function, there has been limited work in this field to examine the basis for diversity in responses of cattle to different dietary challenges.

Although knowledge of associations between the host, the microbiome, and the metabolome is lacking in ruminants, the effects of different diets on the bacterial microbiome and links to the metabolome have been made (Petri et al., 2013). Further, associations to production outcomes have also been made, for example in dairy cattle Jami et al. (2014) demonstrated that the Firmicutes to Bacteroidetes ratio was strongly positively correlated to milk fat (Pearson *R* = 0.72). These authors also describe correlations between other production and efficiency measures and genus abundance. Lima et al. (2015) associated bacterial taxa with milk production and milk components. *Butyrivibrio* spp. and *Prevotellaceae* 2 were some of the bacteria that had the highest positive correlation with milk production (Lima et al., 2015). In beef cattle, Hernandez-Sanabria et al. (2010) linked PCR-DGGE patterns to ruminal fermentation patterns, rumen metabolites, and feed efficiency traits. Roehe et al. (2016) demonstrated host genetic links with microbial methane production in a factorial experiment with crossbred breed types and diet.

The overlapping genetic region between the relative abundance of Actinobacteria, Euryarchaeota, and Fibrobacteres, and A:P is of interest. Diets high in starch induce propionate production which lowers the amount of H_2_ gas produced and also methane production, in comparison to diets with more fiber which tend to increase methane production relative to dry matter intake (Annison and Lewis, 1959; Hungate, 1966). Therefore, we could expect that a high starch diet would lower the abundance of Fibrobacteres, and Euryarchaeota relative to the total microbial population and have a lower A:P. Many members of the phylum Actinobacteria are lactate producers, while others are lactate consumers. Therefore, it is difficult to hypothesize the relationship between this phylum and the A:P as it is likely to depend on the abundance of the different genera that comprise this phylum.

The overlapping regions between Chloroflexi, Tenericutes, and A:P need further investigation, as it is not known whether members of the Chloroflexi phylum are resident bacteria and have any significant role in the rumen (Kim et al., 2011). They are only typically found at low abundances in the rumen and, as sequence datasets are yet to contain any culturable isolates from this phylum, it is difficult to ascertain their metabolism (Kim et al., 2011). The Tenericutes are small prokaryotes that do not contain a cell wall and are therefore resistant to penicillin (Brown, 2011). This may suggest they could also be unaffected by the ionophore and antibiotics (monensin, tylosin, or virginiamycin) given in this study. Mao et al. (2013) showed no change in relative abundance of Chloroflexi and Tenericutes between dairy cattle fed with a subacute acidosis induction diet and controls, but the A:P was decreased in the cattle fed with the subacute acidosis diet. Golder et al. (2014b) showed a tendency toward an increase in Tenericutes in grain-fed heifers, compared to those fed no grain, but there was no effect on relative abundance of Chloroflexi. The A:P was numerically lower in these grain-fed animals, but A:P was not statistically analyzed in the study by Golder et al. (2014b).

Although the Bacteroidetes and Firmicutes phyla are the dominant bacterial phyla in ruminants, these had few marker associations. This could reflect both the diversity of bacterial taxa within these phyla, or perhaps suggests that these phyla comprise a large portion of the core microbiome and are less inherently variable or are preserved as essential to rumen function. The A:P is commonly reported in studies of rumen function and can give an indication of rumen health, when combined with other measures. Hence, it is not surprising that this ratio had more associations than the acetate or propionate measures alone.

It is not surprising that rumen pH had few markers associated as although ruminal pH is commonly used as a diagnostic indicator for acidosis, on its own, point estimates of rumen pH are a poor indicator of acidosis (Golder et al., 2012). Receiver operator curves produced from the dataset of Bramley et al. (2008) that assessed ruminal status of 800 cows from 100 dairies using ruminal, performance, feed, and fecal characteristics showed that ruminal pH measured from ruminal fluid obtained using a stomach tube and rumenocentesis were only moderately sensitive (0.68 and 0.74, respectively) and specific (0.84 and 0.79, respectively) indicators of ruminal acidosis (Golder et al., 2012).

This research, although preliminary, suggests there is a link between host genetics, the microbiome, and physiological responses and may lead to selection toward acidosis resistant cattle. Such selection is likely to be occurring in the field in herds that are consistently feeding diets that have a high risk of ruminal acidosis. In these herds, there is likely to be a forced selection toward acidosis resistant dams' due to voluntary and involuntary culling as a direct or indirect response to ruminal acidosis.

## Conclusions

In summary, our hypothesis that the bovine genome, metabolome, and microbiome are associated in cattle subjected to a non-life threatening, but substantial starch and fructose challenge, with large among animal variation observed is supported by these exploratory observations. These findings assist in understanding the occurrence of inter-animal variation in responses to physiological challenge both in the field and experimentally. They provide a strong rationale for a large field study of natural variation in ruminal acidosis. More research is needed to determine the heritability of microbial constituents and correlated responses to physiological changes. In the future rumen function markers may aid in identification and, therefore, management of animals predisposed to rumen issues.

## Author contributions

HG and IL: conception, design, animal experiment, and metabolome analysis; HG, JT, and IL: host genome analysis and drafting manuscript; HG, SD, and CM: microbial analysis. All authors: interpretation, critical revision, and final approval and accountability.

### Conflict of interest statement

Authors HG and IL were employed by the commercial company Scibus. All other authors declare no competing interests. The authors declare that this study received funding from Scibus and Lallemand Animal Nutrition. Scibus and Lallemand Animal Nutrition were not involved in the study design or collection, analysis, or interpretation of the data.
