# Supplementary material for: Genetic Markers Are Associated with the Ruminal Microbiome and Metabolome in Grain and Sugar Challenged Dairy Heifers
Source: Front Genet. 2018 Feb 27;9:62. doi: 10.3389/fgene.2018.00062 (PMC5835139; doi:10.3389/fgene.2018.00062)
Supplement: Supplementary file 1 [file Table1.docx]

**Supplementary Table 1** **|** Positional candidate gene symbols, gene names and associated marker chromosome and position for associated rumen phenotype

| **Gene symbol** | **Gene name** | **Associated markers^a^** | | **Associated phenotype^b^** |
| --- | --- | --- | --- | --- |
|  |  | **Chromosome** | **Position** |  |
| *EPHB1* | *Bos taurus* EPH receptor B1 (EPHB1) | 1 | 135366450 | Acetate: propionate |
|  |  | 1 | 136079152 | Acetate: propionate |
| *TRAPPC3* | *Bos taurus* trafficking protein particle complex 3 (TRAPPC3) | 3 | 110242807 | Acetate: propionate |
|  |  | 3 | 110293079 | Acetate: propionate |
| *SAMM50, SULT4A1, PARVB, PARVG, PRR5, NUP50, MIR1249, UPK3A, FAM118A, RIBC2, FBLN1, ATXN10, MIR2443, PPARA, CDPF1, TTC36, GTSE1, TRMU, GRAMD4* | *Bos taurus* SAMM50 sorting and assembly machinery component (SAMM50), *Bos taurus* sulfotransferase family 4A member 1 (SULT4A1), *Bos taurus* parvin beta (PARVB), *Bos taurus* parvin gamma (PARVG), *Bos taurus* nucleoporin 50kDa (NUP50), *Bos taurus* microRNA mir-1249 (MIR1249), *Bos taurus* uroplakin 3A (UPK3A), *Bos taurus* family with sequence similarity 118 member A (FAM118A), *Bos taurus* RIB43A domain with coiled-coils 2 (RIBC2), *Bos taurus* fibulin 1 (FBLN1), *Bos taurus* ataxin 10 (ATXN10), *Bos taurus* microRNA mir-2443 (MIR2443), *Bos taurus* peroxisome proliferator-activated receptor alpha (PPARA), *Bos taurus* cysteine rich, DPF motif domain containing 1 (CDPF1), *Bos taurus* tetratricopeptide repeat domain 38 (TTC38), *Bos taurus* G2 and S-phase expressed 1 (GTSE1), *Bos taurus* tRNA 5-methylaminomethyl-2-thiouridylate methyltransferase (TRMU), transcript variant 2, non-coding RNA, *Bos taurus* GRAM domain containing 4 (GRAMD4) | 5 | 114773811 | Acetate: propionate |
|  |  | 5 | 118078218 | Acetate: propionate |
| *MEPE, IBSP, LAP3, MED28, DCAF16, NCAPG, LCORL* | *Bos taurus* matrix extracellular phosphoglycoprotein (MEPE), *Bos taurus* integrin binding sialoprotein (IBSP), *Bos taurus* leucine aminopeptidase 3 (LAP3), *Bos taurus* mediator complex subunit 28 (MED28), *Bos taurus* DDB1 and CUL4 associated factor 16 (DCAF16), *Bos taurus* non-SMC condensin I complex subunit G (NCAPG), *Bos taurus* ligand dependent nuclear receptor corepressor-like (LCORL) | 6 | 38258290 | Acetate: propionate |
|  |  | 6 | 39527071 | Acetate: propionate |
| *GALNTL6* | *Bos taurus* polypeptide N-acetylgalactosaminyltransferase-like 6 (GALNTL6) | 8 | 3922416 | Acetate: propionate |
| *AKT3, SDCCAG8, CEP170, PLD5, EXO1, MAP1LC3C, WDR64, KMO, FH, RGS7* | v-akt murine thymoma viral oncogene homolog 3 (AKT3), serologically defined colon cancer antigen 8 (SDCCAG8), centrosomal protein 170kDa (CEP170), phospholipase D family member 5 (PLD5), exonuclease 1 (EXO1), microtubule associated protein 1 light chain 3 gamma (MAP1LC3C), WD repeat domain 64 (WDR64), kynurenine 3-monooxygenase (kynurenine 3-hydroxylase) (KMO), fumarate hydratase (FH), egulator of G-protein signaling 7 (RGS7) | 16 | 34166299 | Butyrate (m*M*) |
|  |  | 16 | 36630447 | Butyrate (m*M*) |
|  |  | 16 | 36723198 | Butyrate (m*M*) |
| *OXNAD1* | *Bos taurus* oxidoreductase NAD-binding domain containing 1 (OXNAD1) | 1 | 155101934 | Total lactate (m*M*)^c^ |
|  |  | 1 | 155184442 | Total lactate (m*M*)^c^ |
| *NUDCD3, CAMK2B* | *Bos taurus* NudC domain containing 3 (NUDCD3), *Bos taurus* calcium/calmodulin-dependent protein kinase II beta (CAMK2B) | 4 | 77666750 | Total lactate (m*M*)^c^ |
|  |  | 4 | 77766641 | Total lactate (m*M*)^c^ |
| *SMARCAD1, ATOH1* | *Bos taurus* SWI/SNF-related, matrix-associated actin-dependent regulator of chromatin, subfamily a, containing DEAD/H box 1 (SMARCAD1), *Bos taurus* atonal bHLH transcription factor 1 (ATOH1) | 6 | 31611312 | Total lactate (m*M*)^c^ |
|  |  | 6 | 32489075 | Total lactate (m*M*) |
| *C1QTNF7, MIR2448, FBXL5, CD38, FGFBP1, PROM1, LDB2* | *Bos taurus* C1q and tumor necrosis factor related protein 7 (C1QTNF7), *Bos taurus* microRNA mir-2448 (MIR2448, *Bos taurus* F-box and leucine-rich repeat protein 5 (FBXL5),*Bos taurus* CD38 molecule (CD38), *Bos taurus* fibroblast growth factor binding protein 1 (FGFBP1), *Bos taurus* prominin 1 (PROM1),*Bos taurus* LIM domain binding 2 (LDB2), transcript variant 1 | 6 | 115378011 | Total lactate (m*M*)^c^ |
|  |  | 6 | 116755758 | Total lactate (m*M*)^c^ |
| *CARD9* | *Bos taurus* caspase recruitment domain family member 9 (CARD9) | 11 | 103836737 | Total lactate (m*M*)^c^ |
|  |  | 11 | 103896312 | Total lactate (m*M*)^c^ |
| *ALDH1L1, KLF15, CFAP100* | *Bos taurus* aldehyde dehydrogenase 1 family member L1 (ALDH1L1), *Bos taurus* Kruppel-like factor 15 (KLF15), *Bos taurus* cilia and flagella associated protein 100 (CFAP100) | 22 | 61149265 | Total lactate (m*M*) |
|  |  | 22 | 61378199 | Total lactate (m*M*) |
| *DSG3* | *Bos taurus* desmoglein 3 (DSG3) | 24 | 26009491 | Total lactate (m*M*)^c^ |
|  |  | 24 | 26013849 | Total lactate (m*M*)^c^ |
| *EPB41L3, TMEM200C* | *Bos taurus* erythrocyte membrane protein band 4.1-like 3 (EPB41L3), *Bos taurus* transmembrane protein 200C (TMEM200C) | 24 | 39341875 | Total lactate (m*M*)^c^ |
|  |  | 24 | 39800838 | Total lactate (m*M*)^c^ |
| *BACE2, MX2, MX1* | *Bos taurus* beta-site APP-cleaving enzyme 2 (BACE2), *Bos taurus* MX dynamin-like GTPase 2 (MX2), *Bos taurus* MX dynamin-like GTPase 1 (MX1) | 2 | 123152276 | D-lactate (m*M*)^c^ |
|  |  | 2 | 125435419 | D-lactate (m*M*)^c^ |
| *TMEM261, LOC617079, GLDC, UHRF2, IL33, MLANA, PDCD1LG2, CD274, PLGRK* | *Bos taurus* transmembrane protein 261 (TMEM261), *Bos taurus* ATP-binding cassette transporter C4-like (LOC617079), *Bos taurus* glycine dehydrogenase (decarboxylating) (GLDC), *Bos taurus* ubiquitin-like with PHD and ring finger domains 2, E3 ubiquitin protein ligase (UHRF2), *Bos taurus* interleukin 33 (IL33), *Bos taurus* melan-A (MLANA), *Bos taurus* programmed cell death 1 ligand 2 (PDCD1LG2), *Bos taurus* CD274 molecule (CD274), *Bos taurus* plasminogen receptor, C-terminal lysine transmembrane protein (PLGRKT) | 8 | 36781224 | D-lactate (m*M*)^c^ |
|  |  | 8 | 39541982 | D-lactate (m*M*)^c^ |
| *ADGRA1, BNIP3* | *Bos taurus* adhesion G protein-coupled receptor A1 (ADGRA1), *Bos taurus* BCL2/adenovirus E1B 19kDa interacting protein 3 (BNIP3) | 26 | 50680745 | D-lactate (m*M*)^c^ |
|  |  | 26 | 51426365 | D-lactate (m*M*)^c^ |
| *CHMP2B, POU1F1, HTR1F, CGGBP1, C1H3orf38, EPHA3, PROS1* | *Bos taurus* charged multivesicular body protein 2B (CHMP2B), *Bos taurus* POU class 1 homeobox 1 (POU1F1), *Bos taurus* 5-hydroxytryptamine (serotonin) receptor 1F, G protein-coupled (HTR1F), *Bos taurus* CGG triplet repeat binding protein 1 (CGGBP1), *Bos taurus* chromosome 1 open reading frame, human C3orf38 (C1H3orf38), *Bos taurus* EPH receptor A3 (EPHA3), *Bos taurus* protein S (alpha) (PROS1) | 1 | 34903570 | L-lactate (m*M*)^c^ |
|  |  | 1 | 37934858 | L-lactate (m*M*)^c^ |
| *OXNAD1* | *Bos taurus* oxidoreductase NAD-binding domain containing 1 (OXNAD1) | 1 | 155101934 | L-lactate (m*M*)^c^ |
|  |  | 1 | 155184442 | L-lactate (m*M*)^c^ |
| *NUDCD3, CAMK2B* | *Bos taurus* NudC domain containing 3 (NUDCD3), *Bos taurus* calcium/calmodulin-dependent protein kinase II beta (CAMK2B) | 4 | 77666750 | L-lactate (m*M*)^c^ |
|  |  | 4 | 77766641 | L-lactate (m*M*)^c^ |
| *ROR2, NFIL3, AUH, SYK, DIRAS2* | *Bos taurus* receptor tyrosine kinase-like orphan receptor 2 (ROR2), *Bos taurus* nuclear factor, interleukin 3 regulated (NFIL3), *Bos taurus* AU RNA binding protein/enoyl-CoA hydratase (AUH), *Bos taurus* spleen tyrosine kinase (SYK), *Bos taurus* DIRAS family, GTP-binding RAS-like 2 (DIRAS2) | 8 | 87308122 | L-lactate (m*M*)^c^ |
|  |  | 8 | 89061216 | L-lactate (m*M*)^c^ |
| *UBC, SCARB1* | *Bos taurus* ubiquitin C (UBC), *Bos taurus* scavenger receptor class B member 1 (SCARB1) | 17 | 53098878 | L-lactate (m*M*) |
|  |  | 17 | 53755199 | L-lactate (m*M*) |
| *DSG3* | *Bos taurus* desmoglein 3 (DSG3) | 24 | 26009491 | L-lactate (m*M*)^c^ |
|  |  | 24 | 26013849 | L-lactate (m*M*)^c^ |
| *CSNK1D, SLC16A3, FASN, DUS1L, GPS1, DCXR, RAC3, LRRC45, STRA13, ASPSCR1, MIR2346, MYADML2, PYCR1, MAFG, SIRT7, PCYT2, MIR2347, ANAPC11, NYB, ALYREF, ARHGDIA, P4HB, PPP1R27, GCGR, FAM195B, MRPL12, HGS, CCDC137, OXLD1, PDE6G, NPLOC4, FSCN2, ACTG1, MIR3533* | *Bos taurus* casein kinase 1 delta (CSNK1D), *Bos taurus* solute carrier family 16 (monocarboxylate transporter), member 3 (SLC16A3), *Bos taurus* fatty acid synthase (FASN), *Bos taurus* dihydrouridine synthase 1-like (DUS1L), *Bos taurus* G protein pathway suppressor 1 (GPS1), *Bos taurus* L-xylulose reductase-like (DCXR), *Bos taurus* ras-related C3 botulinum toxin substrate 3 (rho family, small GTP binding protein Rac3) (RAC3), *Bos taurus* leucine rich repeat containing 45 (LRRC45), *Bos taurus* stimulated by retinoic acid 13 (STRA13), *Bos taurus* alveolar soft part sarcoma chromosome region, candidate 1 (ASPSCR1), *Bos taurus* microRNA 2346 (MIR2346), *Bos taurus* myeloid-associated differentiation marker-like 2 (MYADML2), *Bos taurus* pyrroline-5-carboxylate reductase 1 (PYCR1), *Bos taurus* v-maf avian musculoaponeurotic fibrosarcoma oncogene homolog G (MAFG), *Bos taurus* sirtuin 7 (SIRT7), *Bos taurus* phosphate cytidylyltransferase 2, ethanolamine (PCYT2), *Bos taurus* microRNA 2347 (MIR2347), anaphase promoting complex subunit 11 (ANAPC11), neuropeptide B (NPB), Aly/REF export factor (ALYREF), *Bos taurus* Rho GDP dissociation inhibitor (GDI) alpha (ARHGDIA), *Bos taurus* prolyl 4-hydroxylase, beta polypeptide (P4HB), *Bos taurus* protein phosphatase 1 regulatory subunit 27 (PPP1R27), *Bos taurus* glucagon receptor (GCGR), *Bos taurus* family with sequence similarity 195 member B (FAM195B), *Bos taurus* mitochondrial ribosomal protein L12 (MRPL12), *Bos taurus* hepatocyte growth factor-regulated tyrosine kinase substrate (HGS), *Bos taurus* coiled-coil domain containing 137 (CCDC137), *Bos taurus* phosphodiesterase 6G (PDE6G), *Bos taurus* oxidoreductase-like domain containing 1 (OXLD1), *Bos taurus* nuclear protein localization 4 homolog (*S. cerevisiae*) (NPLOC4), *Bos taurus* fascin actin-bundling protein 2, *Bos taurus* retinal (FSCN2), *Bos taurus* actin gamma 1 (ACTG1), *Bos taurus* microRNA 3533 (MIR3533) | 19 | 51914513 | Acidosis eigenvalue^d^ |

^a^ Based on a Bonferroni adjusted *P*-value <0.05.

^b^ Rumen samples were collected at 7-day intervals (Pre = preadaptation (day 0), AI = adaptation I (day 7), AII = adaptation II (day 14), and challenge day (day 21). On the challenge day, rumen fluid samples were collected 5, 65,115, 165, and 215 minutes after challenge ration consumption.

^c^ Only present at one sampling time point.

^d^ Derived from discriminant analysis of standardized values for rumen acetate, propionate, butyrate, valerate, iso-butyrate, iso-valerate, ammonia, pH, and D lactate [Bramley, E., Lean, I.J., Fulkerson, W.J., Stevenson, M.A., Rabiee, A.R., and Costa, N.D. (2008). The definition of acidosis in dairy herds predominantly fed on pasture and concentrates. *J. Dairy Sci.* 91**,** 308-321. doi: 10.3168/jds.2006-601].
