# Supplementary material for: Genetic Markers Are Associated with the Ruminal Microbiome and Metabolome in Grain and Sugar Challenged Dairy Heifers
Source: Front Genet. 2018 Feb 27;9:62. doi: 10.3389/fgene.2018.00062 (PMC5835139; doi:10.3389/fgene.2018.00062)
Supplement: Supplementary file 2 [file Table2.docx]

**Supplementary Table 2 |** Positional candidate gene symbols, gene names and associated marker chromosome and position for microbial phyla associated from more than one rumen sampling time point^a^.

| **Gene symbol** | **Gene name** | **Associated markers^b^** | | **Associated phyla^c^** |
| --- | --- | --- | --- | --- |
|  |  | **Chromosome** | **Position** |  |
| **Bacterial phyla** |  |  |  |  |
| *DAZL,PLCL2, TBC1D5, SATB1, MIR2285E-1* | *Bos taurus* deleted in azoospermia like (DAZL), *Bos taurus* phospholipase C-like 2 (PLCL2), *Bos taurus* TBC1 domain family member 5 (TBC1D5), *Bos taurus* SATB homeobox 1 (SATB1), *Bos taurus* microRNA 2285e-1 (MIR2285E-1) | 1 | 155184442 | Actinobacteria |
|  |  | 1 | 158229218 | Actinobacteria |
| *FARSB* | *Bos taurus* phenylalanyl-tRNA synthetase beta subunit (FARSB) | 2 | 111335667 | Actinobacteria |
|  |  | 2 | 111425104 | Actinobacteria |
| *ZNF326, LRRC8D, MIR2904-1, MIR2904-2, MIR2904-3, MIR2887-1, MIR2887-2, MIR2285K-5, LRRC8C* | *Bos taurus* zinc finger protein 326 (ZNF326), *Bos taurus* leucine rich repeat containing 8 family member D (LRRC8D), *Bos taurus* microRNA 2904-1 (MIR2904-1), *Bos taurus* microRNA 2904-2 (MIR2904-2), *Bos taurus* microRNA 2904-1 (MIR2904-3), *Bos taurus* microRNA 2887-1 (MIR2887-1), *Bos taurus* microRNA 2887-2 (MIR2887-2), *Bos taurus* microRNA 2285k-5 (MIR2285K-5), *Bos taurus* leucine rich repeat containing 8 family member C (LRRC8C) | 3 | 52976013 | Actinobacteria |
|  |  | 3 | 53324286 | Actinobacteria |
|  |  | 3 | 53574180 | Actinobacteria |
| *NUDCD3. CAMK2B, YKT6, GCK* | *Bos taurus* NudC domain containing 3 (NUDCD3), *Bos taurus* calcium/calmodulin dependent protein kinase II beta (CAMK2B), *Bos taurus* YKT6 v-SNARE homolog (S. cerevisiae) (YKT6), *Bos taurus* glucokinase (GCK) | 4 | 77666750 | Actinobacteria |
|  |  | 4 | 77766641 | Actinobacteria |
| *MEPE, IBSP, LAP3, MED28, DCAF16* | *Bos taurus* matrix extracellular phosphoglycoprotein (MEPE), *Bos taurus* integrin binding sialoprotein (IBSP), *Bos taurus* leucine aminopeptidase 3 (LAP3), *Bos taurus* mediator complex subunit 28 (MED28), *Bos taurus* DDB1 and CUL4 associated factor 16 (DCAF16) | 6 | 38402326 | Actinobacteria |
|  |  | 6 | 38578930 | Actinobacteria |
| *PGM5, FAM122A, FXN, TJP2, APBA1* | *Bos taurus* phosphoglucomutase 5 (PGM5), *Bos taurus* family with sequence similarity 122A (FAM122A), *Bos taurus* frataxin (FXN), *Bos taurus* tight junction protein 2 (TJP2), *Bos taurus* amyloid beta precursor protein binding family A member 1 | 8 | 45012379 | Actinobacteria |
|  |  | 8 | 45015797 | Actinobacteria |
|  |  | 8 | 45450580 | Actinobacteria |
| *SDR16C5, SDR16C6, PENK* | *Bos taurus* short chain dehydrogenase/reductase family 16C, *Bos taurus* member 5 (SDR16C5), *Bos taurus* short chain dehydrogenase/reductase family 16C, member 6 (SDR16C6), *Bos taurus* proenkephalin (PENK) | 14 | 25284162 | Actinobacteria |
|  |  | 14 | 25329035 | Actinobacteria |
| *GLRZ2, TROVE2, UCHL5, RGS12, RGS1, RGS18* | *Bos taurus* glutaredoxin 2 (GLRX2), *Bos taurus* TROVE domain family member 2 (TROVE2), *Bos taurus* ubiquitin C-terminal hydrolase L5 (UCHL5), *Bos taurus* regulator of G-protein signaling 13 (RGS13), *Bos taurus* regulator of G-protein signaling 1 (RGS1), *Bos taurus* regulator of G-protein signaling 18 (RGS18) | 16 | 12741497 | Actinobacteria |
|  |  | 16 | 12802251 | Actinobacteria |
|  |  | 16 | 13052594 | Actinobacteria |
|  |  | 16 | 13842004 | Actinobacteria |
| *ALDH1L1, KLF15, CFAP100* | *Bos taurus* aldehyde dehydrogenase 1 family member L1 (ALDH1L1), *Bos taurus* Kruppel like factor 15 (KLF15), *Bos taurus* cilia and flagella associated protein 100 (CFAP100) | 22 | 61298585 | Actinobacteria |
|  |  | 22 | 61378199 | Actinobacteria |
| *EPB41L3, TMEM200C* | *Bos taurus* erythrocyte membrane protein band 4.1 like 3 (EPB41L3), *Bos taurus* transmembrane protein 200C (TMEM200C) | 24 | 39341875 | Actinobacteria |
|  |  | 24 | 39800838 | Actinobacteria |
| *EPHB1, ANAPC13* | *Bos taurus* EPH receptor B1 (EPHB1), *Bos taurus* anaphase promoting complex subunit 13 (ANAPC13), transcript variant 1 | 1 | 135607720 | Chloroflexi |
|  |  | 1 | 136278098 | Chloroflexi |
| *HFM1* | *Bos taurus* HFM1, ATP-dependent DNA helicase homolog (*S. cerevisiae*) (HFM1) | 3 | 52209952 | Chloroflexi |
|  |  | 3 | 52231271 | Chloroflexi |
| *MEPE, IBSP, LAP3, MED28, DCAF16* | *Bos taurus* matrix extracellular phosphoglycoprotein (MEPE), *Bos taurus* integrin binding sialoprotein (IBSP), *Bos taurus* leucine aminopeptidase 3 (LAP3), *Bos taurus* mediator complex subunit 28 (MED28), *Bos taurus* DDB1 and CUL4 associated factor 16 (DCAF16) | 6 | 34903570 | Fibrobacteres |
|  |  | 6 | 37934858 | Fibrobacteres |
| *SIK2, C15H11orf1, CRYAB, HSPB2, DLAT, SDHD, PIH1D2, C15H11orf57, TIMM8B, IL18, TEX12, BCO2* | *Bos taurus* salt inducible kinase 2 (SIK2), *Bos taurus* chromosome 15 open reading frame, human C11orf1 (C15H11orf1), *Bos taurus* crystallin alpha B (CRYAB), *Bos taurus* heat shock protein family B (small) member 2 (HSPB2), *Bos taurus* dihydrolipoamide S-acetyltransferase (DLAT), *Bos taurus* succinate dehydrogenase complex subunit D (SDHD), *Bos taurus* PIH1 domain containing 2 (PIH1D2), *Bos taurus* chromosome 15 open reading frame, human C11orf57 (C15H11orf57), *Bos taurus* translocase of inner mitochondrial membrane 8 homolog B (TIMM8B), *Bos taurus* interleukin 18 (IL18), *Bos taurus* testis expressed 12 (TEX12), *Bos taurus* beta-carotene oxygenase 2 (BCO2) | 15 | 22248887 | Planctomycetes |
|  |  | 15 | 22866102 | Planctomycetes |
| *CXADR* | *Bos taurus* coxsackie virus and adenovirus receptor (CXADR) | 1 | 19097191 | Proteobacteria |
| *CHAF1B* | *Bos taurus* chromatin assembly factor 1 subunit B (CHAF1B) | 1 | 150522239 | Proteobacteria |
| *TCTEX1D1* | *Bos taurus* Tctex1 domain containing 1 (TCTEX1D1) | 3 | 77666750 | Proteobacteria |
| *PATJ* | *Bos taurus* PATJ, crumbs cell polarity complex component (PATJ) | 3 | 84391517 | Proteobacteria |
| *THSD7A* | *Bos taurus* thrombospondin, type I, domain containing 7A (THSD7A) | 4 | 19643696 | Proteobacteria |
| *MDFIC, FOXP2* | *Bos taurus* MyoD family inhibitor domain containing (MDFIC), *Bos taurus* forkhead box P2 (FOXP2) | 4 | 53176385 | Proteobacteria |
|  |  | 4 | 55129325 | Proteobacteria |
| *DPP6* | *Bos taurus* dipeptidyl peptidase like 6 (DPP6) | 4 | 117141918 | Proteobacteria |
| *CCDC91* | *Bos taurus* coiled-coil domain containing 91 (CCDC91) | 5 | 81577169 | Proteobacteria |
|  |  | 5 | 81828636 | Proteobacteria |
| *THAP9, LIN5, MIR2447* | *Bos taurus* THAP domain containing 9 (THAP9), *Bos taurus* in-54 DREAM MuvB core complex component (LIN54), *Bos taurus* microRNA 2447 (MIR2447) | 6 | 99559664 | Proteobacteria |
| *GADD45B, LMNB2* | *Bos taurus* growth arrest and DNA damage inducible beta (GADD45B), *Bos taurus* lamin B2 (LMNB2) | 7 | 22449530 | Proteobacteria |
| *SLC22A4, MIR2457, PDLIM4, P4HA2* | *Bos taurus* solute carrier family 22 member 4 (SLC22A4), *Bos taurus* microRNA 2457 (MIR2457), *Bos taurus* PDZ and LIM domain 4 (PDLIM4), *Bos taurus* prolyl 4-hydroxylase subunit alpha 2 (P4HA2), transcript variant 1 | 7 | 23459285 | Proteobacteria |
| *IGFBPL1, LOC507550* | *Bos taurus* insulin like growth factor binding protein like 1 (IGFBPL1), *Bos taurus* uncharacterized LOC507550 (LOC507550) | 8 | 62891563 | Proteobacteria |
| *GALNT16* | *Bos taurus* polypeptide N-acetylgalactosaminyltransferase 16 (GALNT16) | 10 | 81436407 | Proteobacteria |
| *XPO4, EEF1AKMT1* | *Bos taurus* eukaryotic translation initiation factor 3 subunit E (EIF3E), *Bos taurus* R-spondin 2 (RSPO2) | 12 | 36063522 | Proteobacteria |
| *FOXN4* | *Bos taurus* forkhead box N4 (FOXN4) | 17 | 66038728 | Proteobacteria |
| *CHEK2, HSCB, CCDC117* | *Bos taurus* checkpoint kinase 2 (CHEK2), *Bos taurus* HscB mitochondrial iron-sulfur cluster cochaperone (HSCB), *Bos taurus* coiled-coil domain containing 117 (CCDC117), transcript variant 2 | 17 | 70278544 | Proteobacteria |
| *ZNF331, NLRP12* | *Bos taurus* zinc finger protein 331 (ZNF331), *Bos taurus* NLR family, pyrin domain containing 12 (NLRP12) | 18 | 61054591 | Proteobacteria |
| *SEC14L5, NAGPA, ALG1, EEF2KMT* | *Bos taurus* SEC14-like 5 (*S. cerevisiae*) (SEC14L5), *Bos taurus* N-acetylglucosamine-1-phosphodiester alpha-N-acetylglucosaminidase (NAGPA), *Bos taurus* ALG1, chitobiosyldiphosphodolichol beta-mannosyltransferase (ALG1), *Bos taurus* eukaryotic elongation factor 2 lysine methyltransferase (EEF2KMT) | 25 | 4110250 | Proteobacteria |
| *UBTD1, HOGA1, MORN4, PI4K2A, AVPI1, MARVELD1, ZFYVE27* | *Bos taurus* ubiquitin domain containing 1 (UBTD1), *Bos taurus* 4-hydroxy-2-oxoglutarate aldolase 1 (HOGA1), *Bos taurus* MORN repeat containing 4 (MORN4), *Bos taurus* phosphatidylinositol 4-kinase type 2 alpha (PI4K2A), *Bos taurus* arginine vasopressin induced 1 (AVPI1), *Bos taurus* MARVEL domain containing 1 (MARVELD1), *Bos taurus* zinc finger FYVE-type containing 27 (ZFYVE27) | 26 | 18612734 | Proteobacteria |
|  |  | 26 | 18776144 | Proteobacteria |
| *GLRX3, MIR2397* | *Bos taurus* glutaredoxin 3 (GLRX3), *Bos taurus* microRNA 2397 (MIR2397) | 27 | 3496970 | Proteobacteria |
| *LUZP2* | *Bos taurus* leucine zipper protein 2 (LUZP2) | 29 | 20312863 | Proteobacteria |
|  |  | 29 | 20658120 | Proteobacteria |
| *EPHB1* | *Bos taurus* EPH receptor B1 (EPHB1) | 1 | 135366450 | Tenericutes |
| *CLMP* | *Bos taurus* CXADR like membrane protein (CLMP) | 15 | 34322250 | Tenericutes |
|  |  |  |  |  |
| **Archaeal phylum** |  |  |  |  |
| *HERC3, NAP1L5, PYURF, PIGY, HERC5, HERC6, PPM1K, ABCG2, PKD2, SPP1, MEPE* | *Bos taurus* HECT and RLD domain containing E3 ubiquitin protein ligase 3 (HERC3), *Bos taurus* nucleosome assembly protein 1 like 5 (NAP1L5), *Bos taurus* PIGY upstream reading frame (PYURF), *Bos taurus* phosphatidylinositol glycan anchor biosynthesis class Y (PIGY), *Bos taurus* HECT and RLD domain containing E3 ubiquitin protein ligase 5 (HERC5), *Bos taurus* hect domain and RLD 6 (HERC6), *Bos taurus* protein phosphatase, Mg2+/Mn2+ dependent 1K (PPM1K), *Bos taurus* ATP binding cassette subfamily G member 2 (Junior blood group) (ABCG2), *Bos taurus* polycystin 2, transient receptor potential cation channel (PKD2), secreted phosphoprotein 1 (SPP1), *Bos taurus* matrix extracellular phosphoglycoprotein (MEPE) | 6 | 37756880 | Euryarchaeota |
|  |  | 6 | 37913699 | Euryarchaeota |
|  |  | 6 | 38020906 | Euryarchaeota |
| *SLC4A4, GC, ADAMTS3* | *Bos taurus* solute carrier family 4 member 4 (SLC4A4), *Bos taurus* GC, vitamin D binding protein (GC), *Bos taurus* ADAM metallopeptidase with thrombospondin type 1 motif 3 (ADAMTS3) | 6 | 88743637 | Euryarchaeota |
|  |  | 6 | 89104201 | Euryarchaeota |
|  |  | 6 | 89133819 | Euryarchaeota |
| *BMP2K, PAQR3* | *Bos taurus* BMP2 inducible kinase (BMP2K), *Bos taurus* progestin and adipoQ receptor family member 3 (PAQR3) | 6 | 95386878 | Euryarchaeota |
| *TXNDC16, PTGER2* | *Bos taurus* thioredoxin domain containing 16 (TXNDC16), *Bos taurus* prostaglandin E receptor 2 (PTGER2) | 10 | 11605097 | Euryarchaeota |
|  |  | 10 | 11707725 | Euryarchaeota |
| *ERGIC3, SPAG4, CPNE1, RBM12, NFS1, ROMO1, RBM39, PHF20, SCAND1* | *Bos taurus* ERGIC and golgi 3 (ERGIC3), *Bos taurus* sperm associated antigen 4 (SPAG4), *Bos taurus* copine 1 (CPNE1), *Bos taurus* RNA binding motif protein 12 (RBM12), *Bos taurus* NFS1, cysteine desulfurase (NFS1), reactive oxygen species modulator 1 (ROMO1), *Bos taurus* RNA binding motif protein 39 (RBM39), *Bos taurus* PHD finger protein 20 (PHF20), *Bos taurus* SCAN domain containing 1 (SCAND1) | 13 | 65445282 | Euryarchaeota |
|  |  | 13 | 65899057 | Euryarchaeota |
| *ERGIC1, RPL26L1, ATP6V0E1* | *Bos taurus* endoplasmic reticulum-golgi intermediate compartment 1 (ERGIC1), *Bos taurus* ribosomal protein L26 like 1 (RPL26L1), *Bos taurus* ATPase H+ transporting V0 subunit e1 (ATP6V0E1) | 20 | 50680745 | Euryarchaeota |
|  |  | 20 | 51426365 | Euryarchaeota |

^a^ Rumen samples were collected at 7-day intervals (Pre = preadaptation (day 0), AI = adaptation I (day 7), AII = adaptation II (day 14), and challenge day (day 21). On the challenge day, rumen fluid samples were collected 5, 65,115, 165, and 215 minutes after challenge ration consumption.

^b^ Based on a Bonferroni adjusted *P*-value <0.05.

^c^ The units are relative abundance (%).
